# Recent Advances in the Management of Polycystic Ovary Syndrome: A Review Article

**DOI:** 10.7759/cureus.27689

**Published:** 2022-08-04

**Authors:** Shivani Akre, Kapil Sharma, Swarupa Chakole, Mayur B Wanjari

**Affiliations:** 1 Internal Medicine, Jawaharlal Nehru Medical College, Datta Meghe Institute of Medical Sciences, Wardha, IND; 2 Community Medicine, Jawaharlal Nehru Medical College, Datta Meghe Institute of Medical Sciences, Wardha, IND; 3 Community Health Nursing, Jawaharlal Nehru Medical College, Datta Meghe Institute of Medical Sciences, Wardha, IND

**Keywords:** combined oral contraceptive pills, menstrual irregularity, hyperandrogenism, lifestyle interventions, pcos

## Abstract

Polycystic ovarian syndrome (PCOS) is an endocrine disorder. This condition is characterized by chronic anovulation and ovarian dysfunction, unlike other ovulation disorders when the ovaries are non-functional or abnormal. Currently, most therapy is centred on the patient's primary complaint. Treatment focuses on reducing hyperandrogenism symptoms, restoring menstrual regularity, and achieving conception. In treating infertility caused by polycystic ovarian syndrome, letrozole (an aromatase inhibitor) appears to be more successful than clomiphene citrate (an anti-estrogen and a reference infertility drug). When provided by a multidisciplinary team, it can help patients maintain appropriate lifestyle changes, such as reducing body fat, increasing metabolism, and enhancing reproductive health. Compound oral contraceptives are the most common kind of androgen inhibitor and are the preferred therapy for menstrual disruption in PCOS patients who do not want to get pregnant. Weight loss should be prioritized for women with PCOS since a healthy, balanced diet combined with regular exercise can boost metabolism, increase insulin sensitivity, and aid weight loss safely. This will improve their physical health. Other than reproductive symptoms, PCOS symptoms include insulin resistance (IR), metabolic syndrome (MS), and chronic low-grade inflammation. Our understanding of the pathophysiological process, diagnosis, and therapy of PCOS has advanced recently.

## Introduction and background

Polycystic ovarian syndrome (PCOS) is a widely prevalent endocrine disorder in women accompanied by various symptoms and implications. It has been known for decades to have an incidence of 8-13% in all reproductive age groups, respectively [1]. Anovulation and hypothalamic-pituitary-ovarian axis dysfunction characterise PCOS, although it varies from other types of ovulation failure, marked by inadequate ovarian follicle development or reduced gonadotropin production (or both) (not detected by routine examination). PCOS patients are more likely to have endometrial hyperplasia. Insulin resistance (IR), metabolic syndrome (MS), and persistent low-grade inflammation are some of the other reproductive symptoms of PCOS [2]. Our understanding of the pathophysiological process, diagnosis, and therapy of PCOS has advanced recently. In this study, we focus on lifestyle modifications, type 2 diabetes (T2DM) medications, and bariatric surgery as treatment and prevention methods for metabolic comorbidities in PCOS.

## Review

Management of metabolic comorbidities: other treatment options

Modifications in Lifestyle

Over half of all PCOS sufferers are overweight or obese [3], so PCOS patients are primarily recommended to reduce weight since a good, balanced diet combined with regular exercise can raise their metabolism, improve insulin sensitivity, and help them lose weight safely [4]. Patients of PCOS have hormonal imbalances, high blood cholesterol levels, and are obese. It is critical to understand that working out alone will never be enough to help them lose weight. It is more important to have a healthy diet. For Indian women, diet is seldom a priority. A healthy diet should be high in fibre and protein (1 g/kg body weight). A 30% calorie deficit, or 500 to 750 kcal per day (1200 to 1500 kcal per day), should be stated. According to various studies, overweight people can lose weight, and PCOS females with infertility had irregular ovulation and more excellent responsiveness to ovulation induction drugs, resulting in higher pregnancy and live birth rates. According to research, reducing up to 5% of one's initial weight can help restore regular menstruation and boost the reaction to ovulation and reproductive medications [5].

Genetics

Speaking genetically, the substantial connection of PCOS susceptibility variants in the meta-analysis of genome-wide association (GWAS) data using the cardinal PCOS-associated variables, ovulatory dysfunction (OD), hyperandrogenism (HA), and polycystic ovarian morphology (PCOM), backed the idea that various variations can cause PCOS through different mechanisms [5].

Ovulation Inducers

Ovulation inducement is the cornerstone of treatment for infertile PCOS patients who want to become pregnant because 70% of women with PCOS have dysovulation or no ovulation [5].

Clomiphene citrate is a selective estrogen receptor modulator (SERM)

Clomid citrate (CC) is the drug of choice for ovulation induction in polycystic ovarian syndrome in adolescents [6]. By inhibiting estrogen receptors in the hypothalamus, CC works as an anti-estrogen, increasing the pulse width of gonadotropin-releasing hormone (GnRH) in the anterior pituitary as well as an increase in follicle-stimulating hormone production (FSH). Luteinizing hormone (LH) is a hormone that aids in the development of follicles. CC is usually given for five days between the second and fifth days of the period, commencing at 50 mg per day and rising progressively to 150 mg per day. CC can be administered in tandem with metformin for women with PCOS resistant to CC (conditional evidence-based recommendations, moderate-quality evidence). Clomid is responsible for roughly 30% of successful pregnancies; however, 20% of these pregnancies end in miscarriage or stillbirth. Side effects include ovarian enlargement, hyperstimulation syndrome, multiple pregnancies, hot flushes, gas, bloating, and fatigue [7].

Aromatase inhibitors (AI)-letrozole

Aromatase transforms androgens into estrogen. In the third generation, letrozole is the most widely used non-steroidal selective AI for inducing ovulation. Letrozole inhibits ovarian estradiol secretion. The sensitivity of the follicles to FSH rises when the pituitary secretes more FSH, increasing the ovulation rate. This is due to the hypothalamus's release of negative feedback and a short rise in androgens in the ovary [6].

Gonadotropins

Gonadotropin treatment for women with anovulatory PCOS. Patients who have failed first-line oral ovulation stimulation medicines should consider this as a second-line alternative, such as AI and SERM [7].

Insulin sensitizing agents

Insulin secretion and function are altered in people with PCOS. The effects of hyperinsulinemia and insulin resistance on androgen levels in PCOS patients have long been documented. Insulin controls ovarian activity, and excessive insulin levels can harm the ovaries. Muscle cells produce high quantities of androgens in response to excess insulin, delaying follicular development and resulting in the polycystic ovarian morphology characteristic of PCOS. Acanthosis nigricans has long been used to signify insulin resistance. Insulin resistance makes PCOS patients more vulnerable to long-term health issues like type 2 diabetes and cardiovascular disease, both of which can be fatal [7]. As a result, treating insulin resistance with medications and lifestyle modifications is essential for PCOS therapy [8].

Insulin resistance is traditionally indicated by acanthosis nigricans. Insulin resistance in long term can have systemic adverse side effects. As a result, insulin resistance treatment, including drugs and lifestyle changes, is critical for PCOS treatment [9].

Metformin

Metformin is a biguanide medication that has been proven to be both safe and effective. Even though it is still an authorised application, metformin has long been used to treat type 2 diabetes and is one of the most often utilised insulin sensitisers in treating PCOS. Metformin improves insulin sensitivity in peripheral tissues by lowering hepatic glucose production, boosting glucose absorption, and reducing hepatic glucose synthesis. Metformin side effects include nausea, vomiting, diarrhoea, and abdominal distension. PCOS patients are more likely to acquire prediabetes or type 2 diabetes. Obesity can often generate misunderstandings regarding PCOS and type 2 diabetes mellitus (T2DM), despite this apparent relationship. As a result, type 2 diabetes prevention is essential in this population, and metformin therapy has been shown to lower the incidence of type 2 diabetes in patients with high PCOS. Compared to the broader public, PCOS patients have a poor lipid profile, with a decrease in high-density lipoprotein (HDL) and an increase in triglyceride levels, which are critical predictors of cardiovascular complications. As a result, in PCOS, dyslipidemia treatment is critical. Metformin reduces dyslipidemia by directly reducing hyperinsulinemia or altering the liver's free fatty acid metabolism. Metformin has been demonstrated in several trials to significantly affect dyslipidemia, although it did not affect total cholesterol levels. Metformin is prescribed to women with PCOS at a beginning dose of 500-850 mg per day, which can be raised to 2000 mg per day if tolerated. Metformin in higher doses can help people lose weight and improve their lipid profiles, especially if they are obese and have PCOS. Metformin usage over a long period has also been associated with vitamin B12 deficiency. Metformin has mild gastrointestinal side effects that can be prevented by starting with a modest dose of 500 mg and gradually rising to a maximum dose of 1500 mg once a week [8]. The most common symptoms are abdominal pain, diarrhoea, nausea, vomiting, and minor weight loss. Different therapeutic alternatives for metformin-intolerant women with PCOS should be examined due to metformin intolerance and its associated adverse effects.

Inositol

Inositol, a dietary supplement, aids insulin signalling. Its role in regulating PCOS' metabolic and biochemical components is not well known. According to a new study, menstrual periods and ovulation can be improved. Although this recommendation cautions against using Inositol owing to the limited advantages, it also has a low risk of adverse effects and is cheap [9].

Glucagon-like peptide-1 receptor analogue

Potentiation refers to protinogens such glucagon-like peptide1 (GLP1) and glucose-dependent unguided polypeptides (GIP) that boost glucose-dependent insulin release, especially after a meal. Reaction to incretins Insulin resistance, especially type 2 diabetes, is linked to a change in incretin function. Researchers revealed that PCOS patients have lower levels of the hormone incretin in a recent study. As a result, targeting this system as a treatment for type 2 diabetes has become a feasible alternative, with improved glycemic control and weight loss in type 2 diabetes patients. Mimetics is a promising drug that targets a specific metabolic target and can be used to treat PCOS in various individuals [8].

Statins

Dyslipidemia, which is characterized by high LDL-C, triglycerides, and low HDL-C in PCOS women, is a key predictor of cardiovascular risk. As a consequence, improving the lipid profile and, as a result, decreasing the risk of cardiovascular disease would be a successful PCOS therapy. Statins have been shown to help with the treatment of PCOS. A statin (also known as atorvastatin, fluvastatin, pravastatin, rosuvastatin, and simvastatin) is a drug that prevents cholesterol from being made. In mice, the enzyme 3-hydroxy-3-methylglutaryl coenzyme A (HMGCoA) reductase is essential for cholesterol production. HMG-CoA is transformed to mevalonate when this enzyme is inhibited, limiting cholesterol synthesis. In obese women with PCOS, atorvastatin therapy lowered serum malondialdehyde (MDA), an oxidative stress marker. Furthermore, atorvastatin lowers androstenedione and dehydroepiandrosterone sulfate (DHEAS) levels in this group of PCOS women. When compared to a placebo, atorvastatin enhanced serum vitamin D (25(OH)D) in PCOS patients after a 12-week therapy. However, because of its teratogenic potential, It should not be utilized in reproductive-aged young women. until more rigorous evidence is available to establish its efficacy [8].

Antiandrogens

Spironolactone, flutamide, and finasteride are antigens that reduce hirsutism and acne problems in PCOS patients. Individuals with increased lipid levels, which are frequent in PCOS, may benefit from these antigens. In 40 women with hirsutism, for six months, the effects of spironolactone 100 mg, flutamide 250 mg, and finasteride 5 mg were examined [9]. Despite the fact that there were no significant differences between the groups, all three drugs were effective. Spironolactone (25-100 mg twice daily) is the most commonly given antiandrogen due to its safety, availability, and low cost.

Oral contraceptives

In the treatment of PCOS, the major mechanism of action of OCs is to control menstruation. These drugs also reduce hirsutism, acne, and hirsutism by lowering testosterone levels. Estrogen and progestogen combinations are the most common OCs used to treat hirsutism and acne caused by PCOS. In theory, these medications are more successful than prior formulations at treating androgenic symptoms. After six months of OC therapy, most women with hirsutism achieve clinical improvement. The findings also suggest that antigens and OCs might collaborate to generate synergy. Treatment for PCOS should be offered not just to ease symptoms, but also to avoid long-term complications. Doctors frequently prescribe a combination of oral contraceptives and antigens to lower testosterone levels and alleviate symptoms while preserving the endometrium. Dependence on ethinylestradiol and cyproterone acetate should be minimized due to the increased risk of thromboembolic events, and they should not be considered first-line COCP medications [10,11].

Medroxyprogesterone acetate

PCOS patients who are unable to conceive and are not in danger of becoming pregnant, amenorrhea, or irregular uterine bleeding can be treated with medroxyprogesterone acetate (MPA). Ovarian androgen production is suppressed by monthly progestogen therapy, but abnormal endometrial development is not. MPA also improves insulin sensitivity and lipid profile in PCOS patients [12].

Medications to lose weight

Orlistat

Orlistat is a lipase inhibitor that inhibits triglyceride breakdown in the stomach and pancreas, lowering dietary fat absorption. Orlistat is a weight-loss medicine that has been shown to work, but its effectiveness has been questioned. Orlistat treatment showed significant decreases in body weight and blood levels in a study that investigated the effects of orlistat vs metformin treatment on biochemical and hormonal variables in women with PCOS. Androgen levels are greater than metformin levels [13]. Orlistat also decreased total cholesterol, testosterone, and IR markers. Orlistat also lowers blood pressure and, due to its weight-loss benefits, may assist to avoid type 2 diabetes in this high-risk group. Orlistat has been linked to increased lipodystrophy, diarrhoea, stomach pain, and flatulence when taken 120 mg three times a day with meals, it is the recommended dosage. It might also lead to a deficiency in fat-soluble vitamins. While orlistat may be helpful in the treatment of obesity, its effectiveness in controlling the metabolic aspect of PCOS is disputed. When compared to peer controls and non-obesity PCOS patients, visceral adiposity Index (VAI) levels were greater in overweight and/or obese PCOS patients, and were linked with several metabolic and inflammatory parameters [14].

Sibutramine

The appetite suppressor sibutramine is used in conjunction with lifestyle changes to treat obesity. It's a reuptake inhibitor of monoamines. It prevents neurotransmitters including serotonin, norepinephrine, and dopamine from being absorbed [15].

Rimonabant

Rimonabant is a cannabinoid 1 (CB1) receptor blocker used to treat anorexia and obesity. Rimonabant lowered alanine aminotransferase (ALT) and body weight in obese PCOS patients without nonalcoholic fatty liver disease (NAFLD) [16].

Naltrexone/bupropion

Opioid receptor antagonist naltrexone has a high affinity for the u00b5-opiate receptor, this is connected to eating habits. By decreasing dopamine release, naltrexone has been proven in animal tests to lower food intake, consumption, and binge eating behaviour. It was recently authorized by the US Food and Drug Administration (FDA) for the treatment of alcoholism and drug addiction. Antidepressant bupropion can be used to treat depression and smoking cessation. It acts by preventing the reuptake of dopamine. Weight loss was the most common side effect in clinical trials [15]. Despite the fact that none of these medicines has been authorized by the FDA for the treatment of obesity, scientific trials have shown that combining them results in considerable weight reduction. For example, the combination naltrexone/bupropion (N/B), sold under the oral tablet name Contrave, was recently authorized for the treatment of obesity in the United States and Europe. As a result, naltrexone and bupropion may have clinically significant weight-loss benefits on the metabolic component of PCOS. It's unexpected that this guideline now encompasses infertility surgery and antimicrobial pharmacology in regard to fertility therapy. Despite the paucity of data regarding PCOS and fertility, both treatments should be examined. Because of the potential risks to the baby from pregnancy, women who are having surgery should carefully consider postponing conception until they have achieved nutritional stability after losing weight. Neonatal mortality and growth limitation are two issues that need to be addressed. It can also help those with preeclampsia, gestational diabetes, and big gestational age children, as well as comorbidities including type 2 diabetes, hypertension, and dyslipidemia [16]. 

Vitamin D

There is increasing evidence suggesting that PCOS affects the whole life of a woman, can begin in utero in genetically predisposed subjects, manifests clinically at puberty, and continues during the reproductive years [16]. Vitamin D insufficiency or inadequacy affects 45-90% of reproductive-age women. According to research, vitamin D insufficiency was associated with a substantial reduction in ovulation rate, pregnancy rate, and the chance of a live delivery in PCOS women receiving ovarian stimulation for infertility [17-20]. Patients with polycystic ovarian syndrome, ovulation dysfunction, and metabolic disorders may benefit from vitamin D medication. To make firm conclusions on the effect of vitamin D supplementation on female reproductive health, randomized, prospective, and controlled studies are required [21].

## Conclusions

PCOS is a complex hormonal, metabolic, and psychological disorder with numerous clinical presentations. It is one of the most common reasons for infertility. Before contemplating any pharmaceutical options, lifestyle changes should be considered the primary therapeutic prescription for PCOS-related infertility. According to current research, PCOS increases the risk of endometrial cancer in women of all ages, although it has no effect on the risk of ovarian or breast cancer. These findings point to the possibility of gynaecological cancer morbidity as a result of PCOS.

Ovulation stimulation, which is best performed with letrozole, is the next phase, followed by CC. Gonadotropins were the next step for women who had failed first-line oral ovulatory medication. Pregnant women who do not use ovulation stimulants or who are experiencing other infertility issues may benefit from ART. Metformin in combination with CC or gonadotropin and as an adjuvant to IVF ICSI remains the best option for people with RI or hyperinsulinemia. While inositol and vitamin D may be beneficial to one's health, further study and advice are required. There is no clear proof of the efficacy of alternative treatments. Although insulin sensitizers such as metformin have long been used to treat PCOS metabolic dysfunction, newer medicines such as incretin mimetics and SGLT2 inhibitors have proven to be more effective at reducing weight and cardiovascular risk.
